# Assessment and management of cardiovascular complications in eating disorders

**DOI:** 10.1186/s40337-022-00724-5

**Published:** 2023-01-30

**Authors:** Dara Friars, Orla Walsh, Fiona McNicholas

**Affiliations:** 1 Department of Psychiatry, School of Medicine, University College Dublin, Dublin, Ireland; 2 Department of Paediatrics, Children’s Health Ireland (CHI), Temple Street University Hospital, Dublin, Ireland; 3 Lucena Child and Adolescent Mental Health Service (CAMHS), Dublin, Ireland; 4 Department of Psychiatry, Children’s Health Ireland (CHI), Crumlin, Ireland; 5 Present Address: Mount Pleasant, Australia

**Keywords:** Cardiovascular, Assessment, Eating disorders, Anorexia nervosa, Bulimia nervosa, Avoidant/restrictive food intake disorder, Binge eating disorder, Adolescents and young adults

## Abstract

**Background:**

Eating disorders (EDs) are serious conditions predominantly affecting adolescents and young adults (AYAs) and pose a considerable threat to their health and wellbeing. Much of this increased morbidity and mortality is linked to medical compromise, especially cardiovascular abnormalities. Rates of presentation to both community and inpatient medical settings have increased in all age groups following the Covid-19 pandemic and subsequent “lockdowns”, with patients presentations being more medically compromised compared to previous years. This has implications for clinicians with regard to the performance of competent cardiovascular assessments and management of findings.

**Aims:**

This paper is a practical resource for clinicians working with AYAs in whom EDs may present. It will provide a brief summary of the physiological context in which cardiovascular complications develop, systematically outline these complications and suggest a pragmatic approach to their clinical evaluation.

**Methods:**

Relevant literature, guidelines and academic texts were critically reviewed. Conclusions were extracted and verified by a Child and Adolescent Psychiatrist and Adolescent Paediatrician, with suitable expertise in this clinical cohort.

**Conclusions:**

The cardiovascular complications in EDs are primarily linked to malnutrition, and patients presenting with Anorexia Nervosa are most often at greatest risk of structural and functional cardiac abnormalities, including aberrations of heart rate and rhythm, haemodynamic changes and peripheral vascular abnormalities. Other cardiovascular abnormalities are secondary to electrolyte imbalances, as seen in patients with Bulimia Nervosa. More recently defined EDs including Avoidant/Restrictive Food Intake Disorder and Binge Eating Disorder are also likely associated with distinct cardiovascular complications though further research is required to clarify their nature and severity. Most cardiovascular abnormalities are fully reversible with nutritional restoration, and normalisation of eating behaviours, including the cessation of purging, though rare cases are linked to cardiac deaths. A detailed clinical enquiry accompanied by a thorough physical examination is imperative to ensure the medical safety of AYAs with EDs, and should be supported by an electrocardiogram and laboratory investigations. Consideration of cardiovascular issues, along with effective collaboration with acute medical teams allows community clinicians identify those at highest risk and minimise adverse outcomes in this cohort.

## Plain English summary

Eating disorders (EDs) are serious medical conditions predominantly affecting adolescents and young adults (AYAs). They are often associated with considerable threat to the patient’s health and wellbeing, with Anorexia Nervosa (AN) having the second highest mortality rate of all mental disorders. The cardiovascular system, which includes the heart and blood vessels, is vulnerable to damage and may contribute to this. There are significant concerns that rates of EDs have been increasing, especially in adolescents. Following the onset of the Covid-19 pandemic and subsequent “lockdowns”, there has been an increase in numbers of people of all ages attending community and acute medical settings. Furthermore, presentations are considered to be more medically unwell, with patients presenting with lower weights than previous years. This means that clinicians who might be assessing this group need to be competent in cardiovascular assessments of this cohort. The aim of this paper is to discuss the ways the cardiovascular system is affected in AYAs with various types of EDs, especially AN. The paper aims to provide advice to clinicians on how to assess and cardiovascular problems in this group. This practical summary should be useful to clinicians working in mental health community care who may be less familiar with treating potentially serious physical health problems. The most common cardiovascular issues in young people with low weight or weight loss include slow heart rates, low blood pressure, and problems with sudden changes in blood pressure which can result in fainting. Usually these problems resolve as the patient gains weight. However sometimes more serious changes in heart structure and function may occur and persist causing severe medical issues, like heart failure, which partly explains the high death-rate in AN. For this reason it is important that clinicians know how to spot cardiovascular problems in young people with AN and other EDs and provide them with the best medical care as soon as possible.

## Background

Eating disorders (EDs) are serious conditions characterised by marked disturbances in eating behaviour and often associated with distorted body image and extremes of weight. EDs predominantly affect children, adolescents and young adults and pose a considerable threat to their health and wellbeing. They can cause severe psychiatric and medical complications and are known to impact a patient’s quality of life and mortality. Reframing of ED classification over the last decade has led to the emergence of four well defined disorders including more recently defined binge eating disorder (BED) and avoidant/restrictive food intake disorder (ARFID) in addition to anorexia nervosa (AN) and bulimia nervosa (BN) [1].

AN and ARFID are both restrictive eating disorders, whilst BN and BED are not. AN and BN are EDs characterised by the internalisation of the thin ideal and extreme weight-control behaviours [2]. Patients with AN are underweight and malnourished, whereas patients with BN cycle between binging and restricting but maintain a normal BMI [2]. ARFID and BED are more recently recognised diagnoses and are not associated with body image concerns [1]. Rather they are primarily disorders affecting eating behaviour, the former through avoidance and aversion to food and the latter through frequent binge eating without purging [1]. 

All EDs may occur at any age, though ARFID and AN usually first present in late childhood and adolescence, whereas BED and BN are rare in paediatric cohorts [2]. The grouped lifetime prevalence of AN, BN and BED is approximated to be 8.4% for females and 2.2% for males.] [3] AN carries the second highest mortality risk of all psychiatric conditions, second only to illicit substance use disorders (cocaine and opioids) [4]. Over half of deaths occurring in AN are due to medical complications [5]. Cardiovascular abnormalities are common patients with restrictive EDs (AN and ARFID) and reflect the body’s attempt to conserve energy and compensate for lower blood volume and poor nutrition.

Individuals with EDs were recognised to be a risk group during the Covid-19 pandemic and subsequent “lockdowns”. Many countries reported increased traffic to ED support organisations, specifically: the Butterfly Foundation's National Helpline in Australia noted a 116% increase in calls, while the United Kingdom's eating disorder charity, Beat, and Bodywhys in the Republic of Ireland also reported significantly increased referrals [2, 3, 6–8]. Furthermore, a systematic review concluded that there was an increase in symptoms across all ED types, along with an increase in hospital admissions across different countries [9]. 

Pooled estimates across the studies suggested a 48% increase in admissions during the pandemic compared to pre-pandemic [9]. In this review, 36% of studies (n = 19) documented increases in ED symptoms during the pandemic. These increases were in individuals of all ages and documented in all disorders, AN, BED, BN, and Other Specified Feeding or Eating Disorder (OSFED) patients [9]. Additionally studies found individuals were referred with lower weights, shorter duration of illness and higher levels of medical compromise during Covid-19 [10, 11]. These factors emphasise the need for community clinicians to be cognisant of the associated medical risks.

### The cardiovascular system

Heart rate (HR) and blood pressure (BP) are generally well regulated by neural and hormonal mechanisms but fluctuate in response to food and fluid intake, postural changes, physical activity, temperature, and emotional stress [12–14]. At the cellular level, sodium (Na^+^), potassium (K^+^), calcium (Ca2^+^) and magnesium (Mg2^+^) ions play an important role in the generation and transmission of action potentials through the heart ensuring regular cardiac rhythm [15]. 

### Building a body for a lifetime

The onset of AN and other EDs typically occurs in individuals between 13 and 18 years old, while ARFID is often first diagnosed in childhood [16, 17]. Furthermore, it is suggested that the emergence of severe underweight (BMI < 10th centile for age and gender) is more frequent when EDs present in childhood [16]. An understanding of the youth’s developing cardiovascular system is therefore relevant to clinicians managing EDs.

The World Health Organisation describes children as “building a body for a lifetime”. Due to the demands of growth children and adolescents are in an intense anabolic state, with increased demands for energy, oxygen and water [18]. This results in a dynamic physiology and rapid metabolism, in which inadequate nutrition can leave young people vulnerable to impaired development of organs and systems [18, 19]. The child’s cardiovascular system differs to that of an adult, particularly before the age of twelve [20]. 

Related to the anabolic demands of growth, the child’s HR is faster than an adult’s [20]. Children are less able to alter cardiac contractility, and therefore rely on HR to alter cardiac output (CO) according to the body’s needs. HR is therefore an important clinical marker in this group [20]. However, it is important to realise that HR readings outside normal range in children can be normal under certain circumstances. For example, a child may be tachycardic following vigorous exercise, when feeling stressed or when in pain, whilst mild bradycardia may be normal when asleep [21]. Transient increases in HR with inspiration are also normal and represent a phenomenon known as sinus arrhythmia [22].

Children have lower BP than adults due to decreased peripheral resistance [20]. BP gradually increases as a child grows, reaching adult levels during adolescence. In paediatrics, and unlike HR, BP is a less reliable clinical sign of early cardiovascular compromise as children’s efficient compensatory mechanisms allow them to remain normotensive until they have lost 25% of their intravascular volume [20].

Children are also at increased risk of hypothermia due to their large surface area relative to body mass and decreased insulation by adipose tissue. This is clinically significant as hypothermia predisposes to bradycardia, cardiac instability and cardiac arrest [20]. 

## Cardiovascular complications in anorexia nervosa

Anorexia Nervosa (AN) results in weight-loss, malnutrition, starvation, and is associated with a range of medical complications, such that it carries one of the highest mortality risks of all psychiatric conditions. Half of these are due to medical causes, some cardiovascular in origin [5]. Cardiovascular abnormalities are common findings in patients with AN, occurring in up to 87% at some stage of the illness, and reflect the body’s attempt to conserve energy and compensate for a lower blood volume, and poor nutrition [23]. The cardiovascular complications of AN involve structural and functional cardiac abnormalities, aberrations of heart rate and rhythm, haemodynamic changes and peripheral vascular abnormalities [5]. These are systematically summarised in Table 1.

**Table 1:** Summary of cardiovascular complications in anorexia nervosa [5, 24, 25] Adapted from Sachs et al. [5]

Structural and functional cardiac abnormalities	Repolarisation and conduction abnormalities	Haemodynamic abnormalities	Peripheral vascular abnormalities
Decreased left ventricular mass (LVM) Decreased left ventricular systolic function Mitral valve prolapse Pericardial effusions Myocardial fibrosis	Sinus node dysfunction: Sinus* bradycardia, Sinus pauses and Junctional escape rhythms AV conduction delay QTc prolongation and increased QT dispersion (secondary to electrolyte deficiency and medications)	Hypotension* Orthostatic hypotension and tachycardia* Syncope* Autonomic dysfunction	Acrocyanosis Dysregulation of peripheral vasoconstriction and vasodilation
→ Increased risk of sudden cardiac death ←			

*Denotes the most common complications

Many of the cardiovascular complications are mild, asymptomatic and fully reversible with weight restoration. The most common cardiovascular findings among patients with AN are bradycardia and hypotension [5]. Rarely cardiovascular complications in AN can portend serious medical compromise and be life-threatening [5]. Critically ill individuals with AN carry an increased risk of sudden death [5]. 

### Sinus bradycardia and haemodynamic abnormalities

The most common cardiovascular findings among patients with an AN are sinus bradycardia (sinus rhythm with HR < 60 bpm) and hypotension (< 90/60 mmHg in adults, youth cut-offs guided by age and gender described later) [5]. Sinus bradycardia is considered a compensatory adaptation by the starved body to conserve energy and is mediated by increased vagal tone [5]. Sinus bradycardia is an expected finding in AN and is generally well tolerated [5]. Hypotension occurs secondary to increased vagal tone and may also result from poor fluid intake and dehydration, reduced elasticity in blood vessels, and dysregulation of circadian rhythms [14, 25]. Typically hypotension is asymptomatic, but gravity encourages pooling of blood in lower limbs, and the consequential reduced cardiac output coupled with a slower heart rate, may give rise to pre-syncope and syncope [5]. 

Postural orthostatic tachycardia syndrome (POTS) is a disorder of the autonomic nervous system [26]. The patient experiences a number of symptoms due to poor cerebral perfusion upon standing and an increase in HR often over 120 bpm, in the absence of orthostatic hypotension. Orthostatic HR increases in excess of 30 bpm occur, and the patient may experience dizziness, pre-syncope, or exercise intolerance. In some cases, the dramatic orthostatic and heart rate changes seen in patents with low weight and AN have similar features to POTS making the diagnosis difficult. Additionally, like in AN, the typical patient with POTS is female with the onset of symptoms in adolescence. The co-occurrence of POTS in individuals with AN has been acknowledged in clinical services, and to a lesser extent in the literature. However, some consider the presence of AN to be an exclusion factor in diagnosis, such that it remains a contentious issue [5, 27]. 

Autonomic Nervous System (ANS) dysfunction has been explored as a possible cause of the increased risk of cardiac death seen in AN [5]. Heart rate variability (HRV) is generally accepted as a surrogate marker for autonomic activity [5]. In the setting of patients post-myocardial infarction, decreased HRV is a known predictor of mortality and this has led to speculation that a similar association may exist in AN [28, 29]. Whilst the majority of evidence points towards increased HRV in individuals with AN, studies have failed to conclusively delineate a relationship between HRV and sudden death in the condition [30, 31]. It has been suggested that cardiac autonomic control may shift from parasympathetic to sympathetic dominance as duration of AN increases [32]. It is possible that this change in autonomic activity, in tandem with structural and conduction aberrations, may contribute to the risk of sudden cardiac death in AN, though further research is required to explore this hypothesis [5]. 

### Structural and functional abnormalities

Pericardial effusions are identified in approximately 25% of people with AN when screened with echocardiography according to a meta-analysis including 960 patients [33]. All cases identified in the same study were clinically silent [33]. However, there are two case reports of patients with AN and pericardial effusions who required pericardiocentesis [34, 35]. Suggested risk factors for the development of pericardial effusion in AN include lower BMI and reduced triiodothyronine (T3) levels [36]. Myocardial wasting and the loss of pericardial fat resulting in separation of the pericardial layers are proposed mechanisms for the increased incidence of pericardial effusions in AN [36, 37]. Pericardial effusions generally resolve with nutrition [33]. 

AN is associated with a reduction in left ventricular mass (LVM), a reduction in cardiac output (CO) and diastolic dysfunction [33]. Reduced LVM has been observed at an increased rate in AN, even in studies where the controls were thin, suggesting that low-weight alone does not cause cardiac muscle wasting [33]. It is possible that malnutrition and inactivity result in cardiac atrophy, as is the case with skeletal muscle [38–40]. Decreased intravascular volume and decreased peripheral resistance contribute to reduced preload and afterload respectively, which may result in downregulation of LVM and other LV remodelling [33]. Low T3 blood levels have also been implicated in cardiac atrophy in AN, suggesting a possible endocrine basis for the phenomenon [41]. Functionally, these changes to the myocardium manifest as impaired diastolic filling and reduced CO, meaning that the volume of blood expelled from the ventricles with each systole is decreased [33]. Ejection fraction (EF) in patients with AN is typically preserved, indicating that reductions in CO are mediated by reduced stroke volume related to impaired diastolic filling [33]. These changes resolve with refeeding [5]. 

Case reports have detailed incidences of congestive heart failure (CHF) occurring in AN during refeeding, which generally have responded to nutritional restoration and usual medical therapy [24]. Syrup of ipecac is sometimes used by people with AN to induce vomiting [5]. Ipecac is cardiotoxic and can precipitate cardiomyopathy and heart failure. Ipecac induced myopathy is usually reversible with withdrawal of the substance and supportive therapy but can be fatal [42]. Acute cardiac failure has been reported in AN due to Tako-tsubo Cardiomyopathy, also known as stress cardiomyopathy [43–45]. Tako-tsubo cardiomyopathy is characterised by temporary myocardial stunning, typically involving only a part of the left ventricle [43–45]. In AN, triggers may be starvation, malnutrition, hypoglycaemia and re-feeding. Patients may present with acute heart failure, but also ischemic stroke, heart perforation or fatal arrhythmias with sudden cardiac death [45]. 

Myocardial fibrosis has been demonstrated on cardiac MRI and post-mortem studies of patients with AN, and this may contribute to restrictive myocardial dysfunction [33, 46, 47]. Furthermore, myocardial fibrosis may explain regional contraction abnormalities which have been observed in patients in the acute phase of AN [5] Findings from a recent study utilising two-dimensional speckle tracking echocardiography (2DSTE)—derived strain imaging suggests that youth with AN who engage in purging are at increased risk of regional myocardial contraction abnormalities [48]. Clinically this is relevant given the putative relationship between regional myocardial contraction abnormalities, myocardial scarring and risk of malignant arrhythmia [5]. 

Mitral valve prolapse (MVP) is the most common valvular abnormality in AN [5]. A two-dimensional echocardiography study of 43 patients with AN found 37% to have MVP [49]. Valvulo-ventricular disproportion secondary to myocardial atrophy is the likely cause of MVP in AN [50]. Some studies have shown MVP to persist even after weight restoration [50–52]. 

### Repolarisation and conduction abnormalities and sudden death

As discussed, sinus bradycardia is the expected cardiac rhythm in malnourished patients with AN. This occurs secondary to increased vagal tone and reverses with refeeding [5]. Sinus node dysfunction can develop in AN as a result of increased vagal tone, decreased intracardiac glycogen stores, and myocardial atrophy [53]. This can manifest as a junctional escape rhythm, where extra nodal tissues close to the atrioventricular node become the heart’s primary pacemaker to compensate for the failing sinoatrial node. Junctional escape rhythms in AN have been shown to be extinguishable by exercise with reversion to sinus rhythm, suggesting some chronotropic reserve, even in severe AN [5, 53]. Other case reports have detailed more complex sinoatrial blocks occurring in patients with AN [5]. 

The increased incidence of sudden death in AN, along with the structural and conduction-related cardiac abnormalities associated with the condition, have given rise to the suggestion that malignant ventricular arrhythmias may underlie the high mortality risk. QTc prolongation precipitating the polymorphic ventricular arrhythmia, torsade de pointes, has been thought to be a possible mechanism to explain this. However, pre-mortem studies have not shown an increased risk of ventricular tachyarrhythmia in AN [54]. Furthermore, a retrospective study by Frederiksen et al. revealed the risk of prolonged QTc (> 460 ms) as a dichotomous variable was similar between patients with AN and healthy controls.

Current consensus is that QTc prolongation is not inherent to AN, but rather occurs as a sequela of electrolyte deficiency (often hypokalaemia) or medication side effects (see dedicated section and Table 4) [5, 55]. Results from a recent prospective study utilising implantable cardiac monitoring devices in critically underweight patients with AN indicate that clinically significant bradyarrhythmias, particularly sinus pauses, are more common than ventricular tachyarrhythmias in this cohort and represent a more likely cause of sudden cardiac death in AN [54]. Specific data regarding the prevalence of ventricular arrhythmia occurring in AYAs with AN is lacking.

### Peripheral vascular abnormalities

Peripheral vascular abnormalities are frequently seen in patients with AN. Symptoms include cold intolerance, poor peripheral circulation and lower skin temperature, suggesting heat-conserving vasoconstriction. Abnormal vasospasm of the arterioles with compensatory dilation of the post capillary vessels may result in acrocyanosis; a bluish discolouration of the skin most commonly found in the feet and hands, but occasionally in the face. It is often painful, but resolves with weight restoration [5]. 

## Cardiovascular complications in other eating disorders: bulimia nervosa, binge eating disorder and avoidant/restrictive food intake disorder

### Cardiovascular complications in bulimia nervosa

BN is characterised by episodic binge eating coupled with compensatory behaviours, such as post prandial vomiting, fasting and/or substance misuse including laxatives, diuretics, stimulants or thyroid hormone replacement. Weight status reflects the intensity of the compensatory behaviours, and typically ranges from normal to overweight status. Acute cardiovascular complications are typically linked to purging behaviours, however recent longitudinal data suggests that bingeing may also be associated with long-term cardiovascular morbidity and mortality [56]. 

Cardiac arrhythmias represent the highest risk cardiovascular complication related to purging in BN. Electrolyte derangements, often hypokalaemia, occur secondary to self-induced emesis and abuse of diuretics and laxatives. As discussed in relation to AN, hypokalaemia can lead to prolongation of the QT interval which puts individuals at risk of fatal ventricular arrhythmias [57]. Generally, this risk is mitigated by prompt correction of electrolyte deficiencies. However, instances of recurrent ventricular arrhythmia and sudden cardiac death in patients who engage in severe self-induced vomiting have been reported in the literature [58, 59]. Serum electrolyte disturbances seen in BN are summarised in Table 2*.*

**Table 2:** The effect of purging behaviours on serum electrolyte concentrations Adapted from Casiero and Frishman [66]

	Sodium	Potassium	Chloride	Bicarbonate
Diuretics*	↑ →	↓	↓	↑
Laxatives	↑ →	↓	↑ ↓	↑ ↓
Vomiting	↑ ↓	↓	↓	↑
↑ indicates increase; ↓ indicates decrease; → indicates no change				

*Mineralocorticoid receptor antagonists are an exception, increasing serum potassium

Patients with BN may use the emetic drug ipecac to induce vomiting and this can cause direct myocardial injury due to its active compound, emetine. Emetine has specific cardiotoxic properties and has been implicated in the development of myositis and cardiomyopathy [60]. Whilst most evidence suggest that these changes are reversible following discontinuation of ipecac, the threshold for permanent myocardial injury caused by cumulative dosing is not known [61]. Ipecac is no longer commercially available in most jurisdictions due to concerns around its toxicity and potential for abuse. However, it may still be obtained online or via illicit means and thus is an important consideration when determining the aetiology of cardiomyopathy in patients with BN [57]. 

As discussed in relation to AN, HRV is an important marker of autonomic function. A recent systematic review found that individuals with BN also tend to display increased HRV and vagal autonomic dominance [62]. The same study found that autonomic dysfunction is at least partially reversible with standard treatment interventions for BN [62]. Further research is required to delineate the clinical relevance of HRV in BN and whether aberrations may contribute to the risk of cardiac death.

Longitudinal research suggests that women with BN are at increased risk of ischaemic cardiac events, conduction disorders and cardiac death, especially among those requiring frequent hospitalisation [56]. While this is explained in part by purging-related complications discussed above, it is also likely that bingeing has adverse effects on the cardiovascular system. Binge episodes characteristically involve highly processed foods and contain an average of 2415 kcal per episode [63]. The prevalence of hypercholesterolaemia is increased among women with BN (estimates range from 19 to 48%) and it is thought that the binge-restrict cycle is responsible for this derangement which represents a major risk factor for cardiovascular disease [64, 65]. 

Despite the considerable risk of cardiac morbidity and mortality in BN, its medical course is generally less pernicious than that of AN. Patients with BN maintain a normal BMI and thus are spared the cardiovascular complications associated with severe malnutrition as seen in AN [66]. 

### Cardiovascular complications in binge eating disorder (BED)

BED is a common ED and a relatively newly defined diagnosis characterised by regular binge eating episodes during which individuals ingest large amounts of food in a discrete time period, whilst experiencing loss of control over their eating [1, 67]. Disordered eating in BED is similar to BN but individuals with BED do not engage in compensatory behaviours to prevent weight gain. Individuals with BED are therefore commonly in the overweight or obese category and are at risk of metabolic syndrome [67, 68]. Obesity is associated with a plethora of complications, many of which pertain to the cardiovascular system including hypertension, CHF and ischaemic heart disease [68]. Comprehensive discussion of the cardiovascular complications of obesity is beyond the scope of this paper. Given its nascent status as an ED, studies exploring cardiovascular risk specific to BED are limited. It is suggested however that BED may confer increased risk of metabolic syndrome (combination of diabetes, hypertension and obesity) independent of obesity, indicating non-obese individuals with BED could be at increased risk of ischaemic cardiovascular events [68, 69]. Other studies have investigated HRV and autonomic function in BED given their potential as risk markers for cardiac events. Meta-analysis of this data found no difference in resting state HRV between individuals with and without BED [70]. 

### Cardiovascular complications in avoidant/restrictive food intake disorder (ARFID)

ARFID is also a relatively recently defined ED diagnosis, therefore data examining its effect on the cardiovascular system are limited [1]. ARFID shares similarities with AN in that both disorders are characterised by restrictive eating. However symptomatology in ARFID is driven by sensory problems, low appetite and fear of negative consequences related to eating, rather than body image distortion [71]. Patients with ARFID are therefore not focussed on weight loss and this is reflected in an anthropometrically diverse patient population which spans the weight spectrum from underweight to obese [71]. 

Recent clinical guidance from the Royal College of Psychiatrists UK has focused on the acute medical risks associated with underweight status in ARFID [72]. They suggest a general approach to the acute assessment of patients with restrictive EDs, implying that cardiovascular risks linked to low weight and malnutrition may be similar in AN and ARFID. One study which compared medical compromise in individuals with AN and underweight individuals with ARFID found that both groups reported higher incidence of low BP and bradycardia versus healthy controls [71]. However this trend was not reflected in measurements taken during the same study which showed individuals with ARFID to have BP and HR readings closer to those of healthy controls than the lower readings of AN patients [71]. 

Two other retrospective studies examined details from medical admissions of patients with ARFID and found that cardiovascular disturbances were amongst the most common problems necessitating inpatient management [73, 74]. These included bradycardia, prolonged QTc interval and electrolyte derangement (primarily hypokalaemia which poses a cardiac risk as discussed previously) [73, 74]. Further research is required to clarify the nature and extent of cardiovascular system disturbance in ARFID and to delineate the subgroups of ARFID patients most at risk.

## Approach to evaluating the cardiovascular system in eating disorders: history, examination and investigations

A detailed clinical history, thorough physical examination and relevant supportive investigations are core to the evaluation of the cardiovascular system in patients with EDs. Key considerations for the clinician approaching this assessment are outlined below.

### History

A comprehensive psychiatric, dietary and medical history is key, including medications and family history. Clinicians should assess for cardiac symptoms such as chest pain, palpitations, shortness of breath, orthopnoea, paroxysmal nocturnal dyspnoea, dizziness or syncope. A thorough systems review should include details of purging behaviours, fluid intake, degree and rapidity of weight loss and extent of aerobic exercise [22]. The clinical relevance of these findings from the history is outlined in Table 3.

**Table 3:** Points of enquiry in the cardiovascular history and their clinical relevance in patients with eating disorders Adapted from Treasure [22] with additional references noted

Shortness of breath	Although rare, shortness of breath in AN may be a symptom of congestive heart failure (CHF). The presence of orthopnoea, paroxysmal nocturnal dyspnoea, or breathlessness worsened on exertion increases the index of suspicion for CHF [24].
Palpitations	Palpitations are often benign but may indicate arrhythmia, valvular dysfunction, or autonomic dysfunction [25]. Tachycardia at rest is a worrisome sign in AN, often indicating a separate underlying cause, e.g. infection [25]. Arrhythmias and autonomic dysfunction can cause syncope [5].
Chest pain	Mitral valve prolapse is a possible cause of chest pain in patients with AN and there have been case reports of ischaemic heart disease [5, 25]. Mitral valve prolapse related chest pain may be associated with palpitations and dizziness [75]. Chest pain in young people is usually benign and is non-cardiac in 96% of cases, but in individuals with EDs may represent cardiovascular compromise [72, 75]. Non-cardiac chest pain may be musculoskeletal, psychogenic, gastrointestinal or pulmonary in origin [76]. AN may predispose individuals to spontaneous pneumothorax and this should therefore be considered as a rare but serious differential for chest pain among this group [77, 78].
Syncope and presyncope	Discussed in-text under Cardiovascular abnormalities: Risk stratification and how to respond.
Purging behaviours	Frequent vomiting, as well as abuse of laxatives and diuretics, can cause potassium depletion, predisposing to cardiac arrhythmias [79]. Syrup of ipecac is sometimes used to induce emesis among youth with AN. Ipecac is cardiotoxic and increases the risk of cardiomyopathy [79].
Weight history	Cardiovascular complications are more severe with significant weight loss and low BMI [25]. The degree and rapidity of weight loss should be quantified. In the context of weight stability, failure to gain weight at the expected rate may be equally concerning.
Medical history, medication history	The presence of comorbidities increases cardiac risk. Psychotropic medications, including antipsychotics and antidepressants can adversely affect the heart [80].

Clinicians should account for the fact that patients with medical complaints related to AN often attempt to mask their weight loss and conceal their eating disorder, with under-reporting of physical symptoms common [22]. Collateral history from a caregiver or support person is therefore particularly important in this cohort.

### Physical examination

A thorough physical examination should be performed when a patient first presents and subsequently repeated as clinically indicated, including new or changing physical symptoms, rapid weight loss or gain, or a history of under-reporting symptoms [22]. 

Due to the prevalence of body image distortion, sensitivity around physical examination is particularly relevant in AN [22]. Before beginning, clear consent should be obtained and boundaries established around the extent of the examination. An appropriate chaperone should always be present, especially in the setting of opposite-sex examination [22]. 

Examination should include lying and standing measurement of BP and HR, temperature measurement, examination of peripheries, assessment of jugular venous pulse and respiratory rate, and auscultation of the praecordium [22]. Hydration status should be assessed including measurement of capillary refill time (CRT), quantification of urine output and examination of skin turgor and mucous membranes [72]. Regarding postural observations, clarity around the duration of time required to be lying and standing prior to obtaining orthostatic measurements is lacking and differs by services [82]. Variations in time include lying times ranging from 2 to 30 min, with 5–10 min being the most frequently cited [82]. Reading BP on standing also ranges from an immediate reading to as long as waiting 10 min, with 1–4 min being the most commonly cited. It has been argued that identification of immediate changes will be missed if standing BP is delayed beyond 2 min [82]. 

Weight should be measured regularly, and at more frequent intervals in individuals who are critically low weight. All weights, heights and BMI should be plotted on an age and sex appropriate centile chart and ideal body weight calculated [22]. 

### Electrocardiogram (ECG)

Guidelines suggest that a 12-lead ECG should also be obtained in any patient with AN who is losing weight rapidly, medically compromised, or engaging in severe purging behaviours [72, 83, 84]. Typical findings include bradycardia, along with low QRS voltage and/or right axis deviation which suggests reduced LVM [25]. It is essential to assess for QTc prolongation in patients with electrolyte derangements (K^+^ < 2.5 mmol/L, Mg^2+^  < 0.8 mmol/L, and Ca^2+^  < 2.2 mmol/L) and those taking high risk medications [9, 72, 83]. Medications which predispose to QTc prolongation are outlined in Table 4.

**Table 4:** Medications which predispose to QTc prolongation [80, 81] Adapted from Fanoe et al. [80]

Antipsychotics	Antidepressants	Others
Thioridazine	Amitriptyline	Promethazine
Pimozide	Clomipramine	Diphenhydramine
Ziprasidone	Imipramine	Cyclizine
Chlorpromazine	Escitalopram	Hydroxyzine
Haloperidol	Citalopram	Metoclopramide
Olanzapine	Venlafaxine	Ondansetron
Risperidone		Erythromycin

### Other investigations

Blood tests assessing fluid and electrolyte balance are indicated at baseline, on a yearly basis, and more frequently during refeeding and for patients engaging in compensatory behaviours (purging, laxative use) [84]. 

Echocardiography is not routine, but may be considered in the presence of worrying symptoms (e.g. syncope, orthopnoea, dyspnoea), abnormal examination findings (elevated jugular venous pressure, peripheral oedema, abnormal murmurs, pulsus paradoxus), or concerning ECG findings [5]. 

## Cardiovascular abnormalities: risk stratification and how to respond

### Hypotension and orthostatic changes in BP and HR

In adults standing systolic BP measurements of less than 90 mmHg are of concern medically. For patients under 18 years old, systolic readings below the 0.4th centile of the appropriate age and sex-based reference range should alert clinicians to medical risk [72]. Hypotension which is associated with syncope or orthostatic changes indicates impending risk to life without intervention [72]. Aside from syncope, symptoms of hypotension are typically light-headedness and dizziness [85]. 

Orthostatic hypotension is a decrease in BP on moving from lying to standing. For children and adults, medical review is necessary when BP falls by 15 mmHg systolic within 2 min of standing [72]. Orthostatic hypotension is a particularly high risk sign when systolic BP falls by ≥ 20 mmHg [72]. 

Orthostatic tachycardia is an increase in HR on moving from lying to standing. Orthostatic tachycardia is associated with more severe disease and warrants significant clinical concern and medical review [72]. Particular urgency should be given to cases where the postural increase in HR is of more than 30 bpm in adults or 35 bpm in people under 16 years old [72]. Diagnosis of POTS in patients with concurrent malnourishment secondary to an ED may be inappropriate [27]. 

Management of hypotension and orthostatic disturbances should focus on nutritional rehabilitation and rest until postural observations have normalised [72]. The presence of significant hypotension or postural symptoms merits assessment of hydration and volume status [72]. Oral rehydration is preferred but in patients showing signs of hypovolaemia (tachycardia, inappropriately normal HR) intravenous (IV) fluid therapy may be necessary [72]. Where required and with very careful monitoring, a 10 ml/Kg bolus of 0.9% Normal Saline is recommended for hypovolaemic patients, with further IV fluid management guided by senior medical staff accounting for electrolyte requirements [72]. 

Orthostatic hypotension typically resolves slower than bradycardia, and resolves when weight has been restored to 80% of ideal body weight (IBW), where %IBW is defined as the percentage of median weight for height, age, and gender using the National Centre for Health Statistics (NCHS) tables [86].

### Syncope

Syncope (fainting) and presyncopal symptoms (dizziness/light-headedness) are common in AYAs with EDs and are concerning as they may reflect cardiovascular instability [72]. Any patient experiencing recurrent syncope should be treated with a high level of concern and assessed medically [72]. Syncope is a particularly high risk sign when it is associated with significant postural drops in BP (> 20 mmHg) or profound bradycardia (< 40 bpm) as discussed elsewhere [72]. Patients experiencing pre-syncopal symptoms alone and without orthostatic changes in BP or HR are less concerning but require ongoing monitoring [72]. 

### Sinus bradycardia and other bradyarrhythmias

Sinus bradycardia (< 60 bpm) is the most common heart rhythm found in underweight AYAs with EDs [72]. Sinus bradycardia is often considered a compensatory adaptation to conserve energy in the context of malnutrition, it is generally well tolerated and resolves with refeeding [5]. In malnourished youth with AN, a pseudo normal HR within the expected range may in fact indicate a masked tachycardia which could reflect a compensatory response to hypovolemia, infection or anaemia [25, 72]. Despite sinus bradycardia generally being well tolerated in AN, a very low HR is of clinical concern [72]. A HR < 40 bpm indicates cardiac instability and merits urgent medical review [72]. 

A12-Lead ECG should be performed in patients with HR < 50 bpm in order to confirm sinus bradycardia and exclude other arrhythmias [72]. Other bradyarrhythmias include junctional escape rhythms and heart block which should be treated as high-risk, including referral to acute hospital services for specialise management and cardiac monitoring [72]. Bradyarrhythmia occurring in restrictive EDs should not be treated routinely with atropine or other chronotropic drugs [72]. Even in cases of profound bradycardia, cardiac pacing is generally not required [53]. Reversion to sinus rhythm is typically achieved with refeeding and where required, rewarming [53]. 

### QT interval aberrations

The QT interval prolongs at slower heart rates and shortens at faster heart rates [80]. Measurement of the QT interval is therefore normalised or “corrected” to a heart rate of 60 bpm known as the corrected QT interval (QTc) [80]. Use of automated QTc readings generated by the ECG machine may be feasible where the ECG is otherwise normal [80]. Given the prevalence ECG changes in EDs, clinicians involved in the care of this cohort must be able to measure QTc manually [72, 80]. Manual calculation of QTc is usually carried out with the Bazett formula (QTcB) or the Friderica formula (QTcF). Both formulae are shown below in Table 5. QTcB is the simplest formula to use but overcorrects the QT interval at faster rates and under corrects at slower rates [80]. This is pertinent for clinicians treating patients with restrictive EDs given the ubiquity of vagally mediated bradycardia among this group. Due improved accuracy, QTcF is recommended for use in cases where heart rate falls outside the range of 60–80 bpm, particularly at faster rates [80].

**Table 5:** Formulae for the calculation of the corrected QT interval [80]

Bazett formula for corrected QT interval	Friderica formula for corrected QT interval
$QTcB = \frac{{QT\;{\text{int}} erval\;in\;\sec onds}}{{\sqrt {Cardiac\;cycle\;in\;\sec onds} }} = \frac{QT}{{\sqrt {RR} }}$	${\text{QTcF}} = \frac{{{\text{QT}}\;{\text{ interval}}\;{\text{ in}}\; {\text{seconds}}}}{{\sqrt[3]{{{\text{Cardiac}}\;{\text{ cycle}}\;{\text{ in}}\;{\text{ seconds}}}}}} = \frac{{{\text{QT}}}}{{{{{\text{RR}}}}}}$

Patients with EDs presenting with QTc prolongation should be viewed with concern given the associated risk for torsade de pointes, a form of polymorphic ventricular tachycardia [5, 72]. In adults QTc interval > 450 ms in females and > 430 ms in males is prolonged [72]. QTc interval is generally longer in youth compared to adult populations, with QTc > 460 ms for females under 18 and > 450 ms for males under 18 deemed prolonged [72]. Patients presenting with QTc interval above these limits merit medical assessment and discussion with cardiology [72].

Numerous studies, including a cohort study of over 1000 adults, have shown that QTc prolongation is not intrinsically associated with EDs (5,87) [5, 87]. Rather, it reflects electrolyte derangements (K^+^ < 2.7 mmol/L, Mg^2+^  < 0.8 mmol/L, and Ca^2+^  < 2.2 mmol/L) or medication side-effects (see Table 4) [5]. Clinicians should also consider congenital prolonged QT syndrome, which is often associated with T wave changes, as a possible underlying cause in these cases [72]. The approach to management of long QTc in patients with EDs centres around electrolyte correction and removal of offending medications [5, 72]. 

QT dispersion is the difference between the longest raw QT interval and the shortest QT interval on the 12-lead tracing [5]. QT dispersion values of > 100 ms portend significant risk of cardiac morbidity and mortality [57]. Adolescents in the acute phase of AN are at risk of increased QT dispersion and where identified should be treated as high cardiac risk [5]. 

### Structural cardiac abnormalities

Significant weight loss occurs in AN and leads to a loss of body fat and muscle, which also impacts the myocardium [57]. Relatively common structural abnormalities include reduced left ventricular mass (LVM) and mitral valve prolapse [5]. Decreased LVM is reflected in the ECG as low-voltage QRS and right-axis deviation [5]. These changes may result in reduced CO and rare cases of congestive heart failure (CHF) have been reported. However, left ventricular ejection fraction is typically preserved, with cardiac structure and function usually fully restored on refeeding [5]. 

Pericardial effusions occur frequently in AN (25%), but are generally clinically silent, incidental findings that resolve with refeeding [33]. However, two cases of effusions requiring pericardiocentesis occurring in AN have been reported in the literature [34, 35]. The presence of pulsus paradoxus, i.e. a drop in BP > 10 mmHg with inspiration, suggests patients are at risk of complications from pericardial effusion and merits prompt medical investigation with echocardiography [5]. 

### Peripheral vascular abnormalities

Peripheral vascular abnormalities are frequently seen in patients with AN [5]. Patients complain of cold intolerance, have poor peripheral circulation and lower skin temperature, suggesting heat conserving vasoconstriction [5]. Vasospasm of arterioles with compensatory dilation of the postcapillary venules results in acrocyanosis; a bluish discolouration of the skin at peripheries. [5] This may be painful but resolves with weight restoration [5]. Peripheral vascular abnormalities are usually not thought to pose a risk of tissue damage but do seem to correlate with severe malnutrition [5]. These presence of such findings in a malnourished patient with an ED are generally explained by their physiological context and additional diagnoses such as Raynaud’s disease are likely unwarranted [72]. 

## Conclusion

Eating Disorders (EDs) are an increasingly common source of morbidity in AYAs, with cardiovascular complications a main contributor to their medical burden. Adolescents are physiologically different to adults meaning that their bodies respond uniquely to the stress of undernourishment caused by EDs. The cardiovascular complications involve structural and functional cardiac abnormalities, aberrations of heart rate and rhythm, haemodynamic changes and peripheral vascular abnormalities. These complications are generally a direct result of weight loss and malnutrition, and resolve with weight gain and therefore treatment is nutritional rehabilitation. Although most of the abnormalities identified will reverse with restoration of nutrition, and cessation of ED behaviours, a small number may be linked to cardiac deaths.

Secondary cardiovascular complications can also occur in EDs due to electrolyte derangement caused by purging and medication side effects. Compared to AN and BN, more recently recognised EDs including Avoidant/Restrictive Food Intake Disorder and Binge Eating Disorder are likely associated with distinct cardiovascular complications though further work is necessary to clarify their nature and severity. A detailed clinical enquiry accompanied by a thorough physical examination is imperative to ensure the medical safety of patients, and may be supported by ECG and laboratory investigations where indicated. Community healthcare providers should have a low threshold to refer for medical assessment in the case of concerning abnormalities as outlined in this article. Diligent consideration of cardiovascular issues along with effective collaboration with medical teams allow community clinicians identify those at highest risk and minimise adverse outcomes.

## Data Availability

Not applicable.
